# Validity and reliability of the Short Physical Performance Battery (SPPB): a pilot study on mobility in the Colombian Andes.

**Published:** 2013-09-30

**Authors:** José Fernando Gómez, Carmen-Lucía Curcio, Beatriz Alvarado, María Victoria Zunzunegui, Jack Guralnik

**Affiliations:** 1 Research group in Gerontology and Geriatrics. University of Caldas. Manizales, Colombia.; 2 Department of Community Health and Epidemiology, Queens University, Kingston, Ontario, Canada.; 3 Department of Social and Preventive Medicine Faculty of Medicine, University of Montreal, Research Center of the University of Montreal Hospital Centre (CRCHUM) Quebec, Canada; 4 Department of Epidemiology and Public Health. University of Maryland, School of Medicine, Baltimore, MD, USA.

**Keywords:** SPPB, aging, reliability, validity, validation studies, disability, Colombia

## Abstract

**Objectives:**

To assess the validity (convergent and construct) and reliability of the Short Physical Performance Battery (SPPB) among non-disabled adults between 65 to 74 years of age residing in the Andes Mountains of Colombia.

**Methods:**

Design Validation study; Participants:

150 subjects aged 65 to 74 years recruited from elderly associations (day-centers) in Manizales, Colombia. Measurements: The SPPB tests of balance, including time to walk 4 meters and time required to stand from a chair 5 times were administered to all participants. Reliability was analyzed with a 7-day interval between assessments and use of repeated ANOVA testing. Construct validity was assessed using factor analysis and by testing the relationship between SPPB and depressive symptoms, cognitive function, and self rated health (SRH), while the concurrent validity was measured through relationships with mobility limitations and disability in Activities of Daily Living (ADL). ANOVA tests were used to establish these associations.

**Results:**

Test-retest reliability of the SPPB was high: 0.87 (CI95%: 0.77-0.96). A one factor solution was found with three SPPB tests. SPPB was related to self-rated health, limitations in walking and climbing steps and to indicators of disability, as well as to cognitive function and depression. There was a graded decrease in the mean SPPB score with increasing disability and poor health.

**Conclusion:**

The Spanish version of SPPB is reliable and valid to assess physical performance among older adults from our region. Future studies should establish their clinical applications and explore usage in population studies.

## Abstract (es)

**Objetivos:**

Evaluar la validez convergente y de constructo, y la confiabilidad de la Batería Corta de desempeño físico (Short Physical Performance Battery -SPPB-) entre ancianos no discapacitados entre 65 a 74 años, en los Andes Colombianos.

**Métodos:**

150 ancianos entre 65-74 años reclutados de los centros-día de la ciudad de Manizales, Colombia. Las pruebas del SPPB: equilibrio, caminar 4 m. y el tiempo para incorporarse 5 veces de una silla se aplicaron a todos los participantes. La fiabilidad fue medida con un intervalo de 7 días entre las evaluaciones, y analizada con Anova de medidas repetidas. La validez de constructo fue evaluada mediante análisis factorial y relaciones entre el SPPB con la función cognoscitiva, síntomas de depresión, auto-percepción de salud, y factores sociales; y la validez convergente mediante asociaciones con las limitaciones de movilidad y discapacidad en las Actividades de la Vida Diaria (AVD), para estos últimos análisis se usaron Test de ANOVA.

**Resultados:**

La fiabilidad del SPPB test-retest fue muy buena, 0.87 (IC95%: 0.77- 0.93). Las tres pruebas del SPPB resultaron en un solo factor. El SPPB mostró una relación con limitación en la marcha y para subir escaleras, así como con los indicadores de discapacidad, función cognoscitiva y depresión. Existe una disminución gradual del puntaje de SPPB al aumentar la discapacidad y la mala auto-percepción de salud.

**Conclusión:**

La versión en español de la Batería Corta de Desempeño físico (SPPB) demostró ser válida y confiable para evaluar el desempeño físico en ancianos en los Andes Colombianos.

## Introduction

The Short Physical Performance Battery (SPPB) is one of the most commonly used instruments for measuring physical performance in population studies of aging^1^. The SPPB consists of three subtests: a hierarchical test of balance, a short walk at usual pace and standing up from a chair five times consecutively. Low scores on the SPPB have a high predictive value for a wide range of health consequences including disability in Activities of Daily Living (ADLs)^2^ ^,^ ^3^, loss of mobility^4^, disability^2^ ^,^ ^5^, hospitalization^6^, duration of stay in the hospital^7^, admission to nursing facilities^5^, and death^8^ ^-^ ^11^. The SPPB can be safely used to assess functional capacity in outpatient and clinical settings^12^. Also, it predicts the risk of disability among acutely ill older patients who have been hospitalized^11^ ^,^ ^13^. The reliability of the SPPB for use with elderly populations in the United States is high^2^ and its sensitivity to changes in functional capacity over time has been corroborated^14^ ^,^ ^15^.

Although the SPPB is an objective measure of physical performance less influenced by culture, education and language levels than the self-reported measures of function and disability, there is a need to assess their validity and reliability among several populations before adopting its widespread use in everyday practice. Recently the authors of this article conducted a validation study among elderly populations in two different social and cultural contexts: among the Brazilian elderly with very low socioeconomic status, and with elderly Canadians of high levels of education and income. Notwithstanding the socioeconomic and cultural differences, the study showed high reliability and convergent validity of the battery in both contexts. Thus, these results suggest that the battery is an objective measurement of physical performance that is less influenced by culture, educational level and language than are measures of self-reported function and disability^16^.

The SPPB has not been used in Colombian populations, and the Spanish language version has primarily been used among elderly Mexicans and Mexican Americans^12^ ^,^ ^17^. Reference values have also been established as has its reliability and validity for the Spanish speaking elderly^18^ ^,^ ^19^. Therefore, before considering the introduction of the SPPB in clinical practice or use in aging research in Colombia, the reliability and validity of the battery must be verified.

The objectives of this study were to evaluate: 1) the reliability of the SPPB by measuring at two different test times, 2) convergent validity, relating the SPPB with similar health dimensions, such as the presence of mobility limitations and disability in ADLs in physical and instrumental areas, and 3) construct validity by examining the factorial structure and relating the SPPB with different health dimensions related to mobility, such as self-perceived health, mental function and cognitive function as well as educational level, income and gender. The study was conducted with a sample of elderly community members in the Colombian Andes as a pilot study for an international investigation of mobility and gender, i.e., International Mobility and Aging Study (IMAS).

## Materials and Methods

### Participants: 

a convenience sample of 150 subjects between the ages of 65-74 years was recruited from day-centers in Manizales, Colombia, a city of 400,000 inhabitants in the coffee producing region of the Colombian Andes.

Inclusion criteria for participants were: 1) between 65 and 74 years of age, and 2) not having severe disability in ADLs. Severe disability in ADLs was defined as the inability to perform any of the following activities without the assistance of another person: bathing, getting out of bed, eating and using the toilet. Those who reported difficulties but could do the above noted activities were included in the study. The elderly who had cognitive impairment as noted by four or more errors on the orientation scale of the Prueba Cognitivo de Leganés (PCL) ^20^ (Leganés Cognitive Test), were also excluded.

The Ethics Committee of the University of Caldas approved the study and prior informed consent was obtained from all study participants.

### Data collection:

the interviewers, staff from the area of health, were trained using the same basic curriculum as in the videos, instructions for protocols and original formats. The three battery tests, the scoring system and instructions are clearly explained on a video available on the web (http://www.grc.nia.nih.gov/branches/ledb/sppb/). To evaluate the reliability of the SPPB, data were collected at community centers on two occasions over a period of 5-7 days, with a subsample of 39 participants.

### Short Physical Performance Battery (SPPB)

The SPPB is composed of three tests: a hierarchical assessment of standing balance, a short walk at the usual elderly pace, and standing five times from a seated position in a chair^5^. For balance, participants were asked to remain standing with their feet as close together as possible, then in a semi-tandem position (the ankle of one foot behind the joint of the other foot and finally in a tandem position (ankle of one foot directly behind the other foot and touching it). Each position had to be held for 10 seconds. For gait speed, the time required to travel 4 m at a usual pace was measured. This test was repeated twice and the analysis used the shorter time of the two. For the standing test from a chair, participants were asked to stand and sit in a chair five times as quickly as they could with arms crossed over the chest. This test was performed only after the elderly person demonstrated their ability to stand without using their arms.

Each test was scored from 0 (worst performance) to 4 (best performance): for the balancing test according to a hierarchical combination of performance on the 3 components of the test and a score of 0 for the other 2 tests was assigned to those who did not complete or attempt the task, and scores of 1-4 on the basis of time spent. Additionally, a total score was obtained for the entire battery that was the sum of all 3 tests and varied between 0 and 12^1^ ^,^ ^5^. More details of the administration of this battery and the related published articles may be obtained on the SPPB website 

### Other measures

To evaluate convergent validity the distribution of SPPB (total and each individual test) was compared with the self-report measures of mobility limitations and disability. The number of mobility limitations was calculated with items from the Nagi scale. Participants reported the degree of difficulty in pushing a large object such as an arm chair, carrying a weight of 5 kg, climbing a flight of stairs, walking 400 m and kneeling or squatting. Disability was measured by the number of problems reported in the following ADLs: bathing, dressing, getting out of bed, eating, using the toilet and walking in the room. An additional variable was constructed to evaluate the ranking of mobility disability: a) intact mobility (without Nagi limitations and without difficulties in ADLs); b) mobility limitation (any limitation in Nagi but no difficulty in ADLs, and c) disabilities to perform ADLs (with or without Nagi limitations). To assess construct validity, the SPPB battery was compared with perceived health, cognitive level, depression and socio-demographic factors^16^. Self perceptions of health were assessed with a single question: how would you evaluate your current health? The responses included very good/excellent, good, fair, poor or very poor.

#### Cognitive function

was assessed using the PCL that was originally developed by Spanish researchers as a cognitive screening scale for populations with low educational levels^20^. Scores on this instrument range from 0 to 32 points with higher scores reflecting adequate cognitive functioning^20^. Depressive symptoms were assessed with the Depression Scale from the Center for Epidemiological Studies (CES-D) that consists of 20 items and is applied by self-report. Total scoring totals ranged from 0 to 60. The scale is often dichotomized: a score ≥16 indicates a strong likelihood of depression, and <16 are likely to be without depression.

A structured questionnaire was used to obtain information about age, sex, educational level and perception of income. Educational level was measured as the number of years of formal education ​​(range 0-20). Insufficient income was assessed with a single question: to what degree does your monthly income meet your monthly needs? The answer had four options: very well, so-so, not much and not at all. The last two were categorized as insufficient income.

### Statistical analysis

A descriptive analysis was completed (frequency distribution, means and standard deviations). A within group correlation coefficient was calculated by using an ANOVA test for repeated measures to analyze intra-observer, test-retest reliability. The sample size was sufficient to obtain a correlation close to 0.8 for a continuous measurement of the SPPB score. A minimum acceptable level of reliability was established at 0.60, with a 95% confidence level. Additionally, to assess the construct validity a confirmatory factor analysis was completed to verify a single factor structure of the three tests that make up the SPPB. Finally, to evaluate the construct and convergent validity, an analysis of variance was used to calculate the average SPPB total score along with its three tests, according to socio-demographic variables, functional capacity and health (self perceptions of health, cognitive function and depression). The analysis was performed with SPSS(r) version 18.

## Results

### Description of the study population

Table 1 shows the characteristics of all respondents. The average age was 69.1 years (SD 6.4). The sample consisted mostly of elderly with low levels of education, inadequate incomes, and high levels of functional and cognitive capacity but with low levels of disability (14% of the sample reported some difficulty with ADLs).

**Table 1:**
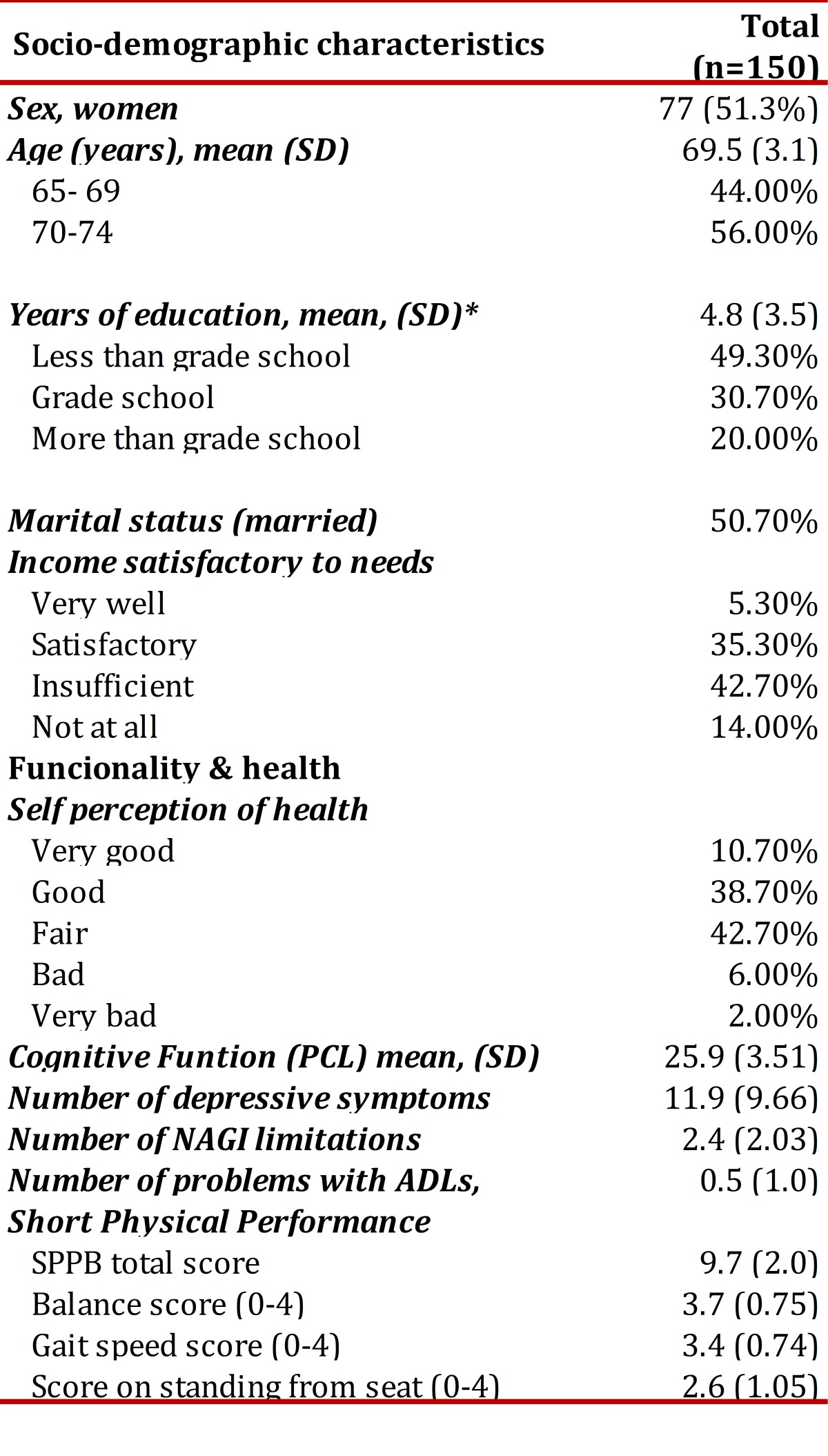
Characteristics of the population studied

The averages of the total battery score for the SPPB and its component tests of balance, gait and standing up from a chair, showed high levels of functioning in the study population. 83% were able to carry out the semi-tandem and tandem tests. The average time for the gait speed test in the sample was 0.79 meters/second (SD 0.19) for the first attempt, and 0.83 m/s (SD 0.18) for the second attempt (range 0.24-1.6). A gait speed of 0.84 m/s corresponds to 10 points on the SPPB and a speed of 0.6 m/s corresponds to 6 points on the SPPB. The average time for standing up from a chair was 13.87 seconds (SD: 5.48). Table 2 shows the distribution of scores for each of the battery tests. As the distribution shows, low scores for SPPB tests (0-2) were observed in 8.6% of participants for the balancing test, 13.3% for gait speed and 44% for standing up from a chair.

**Table 2:**
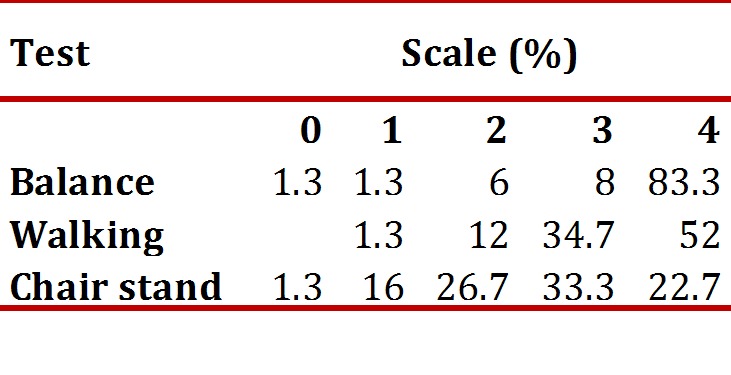
Percentage distribution of the scores for each test of the physical performance battery (SPPB)

### Test reliability

The within group correlation for the total battery score was high, i.e. 0.87 (95%CI: 0.76 to 0.93). The reliability was high for the components of gait speed, 0.92 (95%CI: 0.85 to 0.96) and for standing up from a chair, 0.75 (95%CI: 0.50 to 0.86) and less, although acceptable, for the balancing component, 0.64 (95%CI: 0.31, 0.81).

### Construct validity

 presents the results of the factor analysis. The three SPPB tests resulted in a single factor with high factor loadings. Table 3 shows the extent that participants reported their health as poor or very poor and fair had lower SPPB scores, 9.0 (SD 2.6) and 9.5 (SD 2.1) respectively, with the best scores for the elderly for those that reported their health as excellent, 10.8 (SD 1.1) (*p* <0.05). There was a tendency to speed up and get up from a chair more quickly for those with higher scores than those who reported better self-perceived health (*p*= <0.05, data not shown). No significant relationships were found between the SPPB (total score) and sex, age, education and income.

**Table 3:**
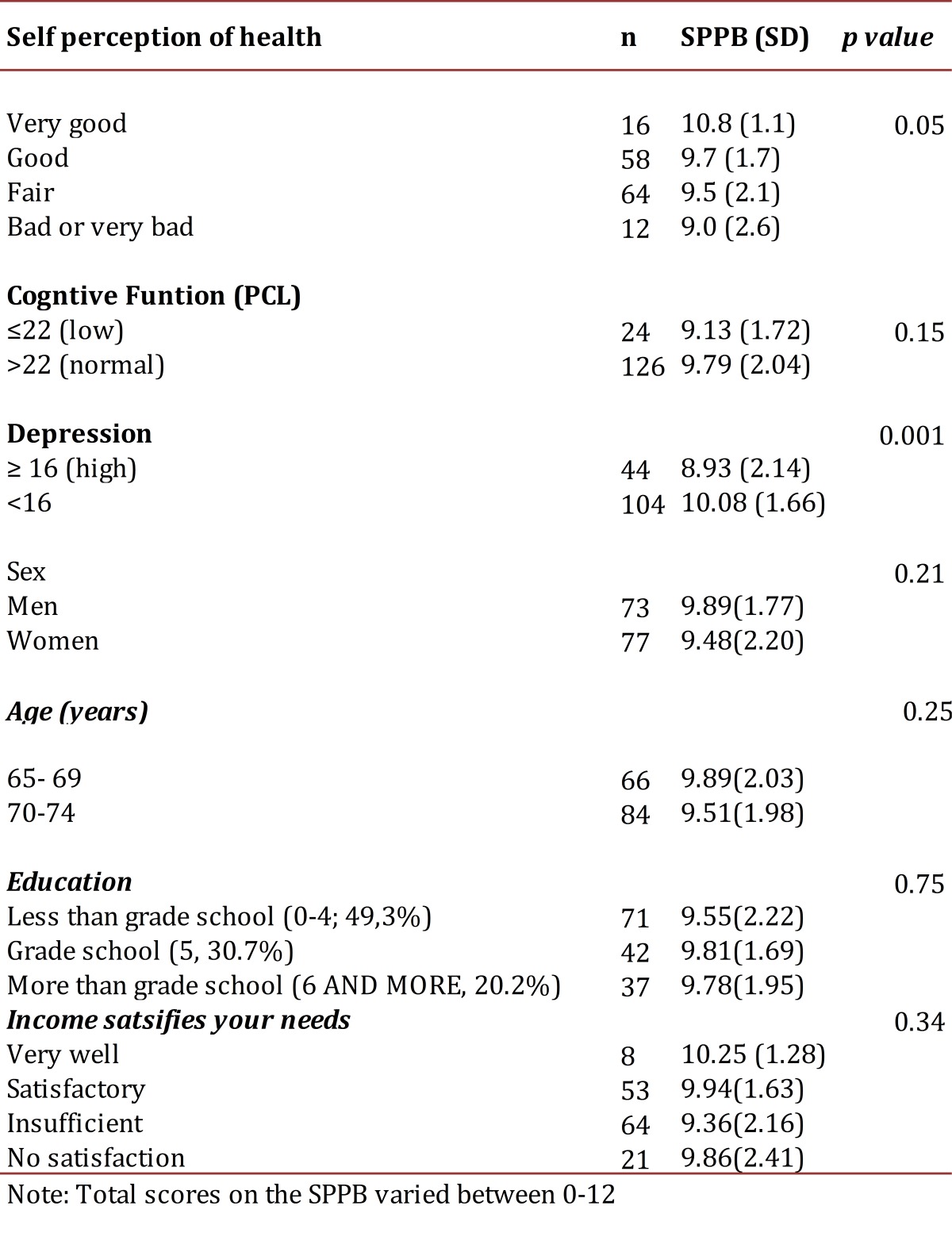
Average score on the SPPB battery according to self perception of health, function, cognitive functioning and depression

### Convergent validity

**Table 4:**
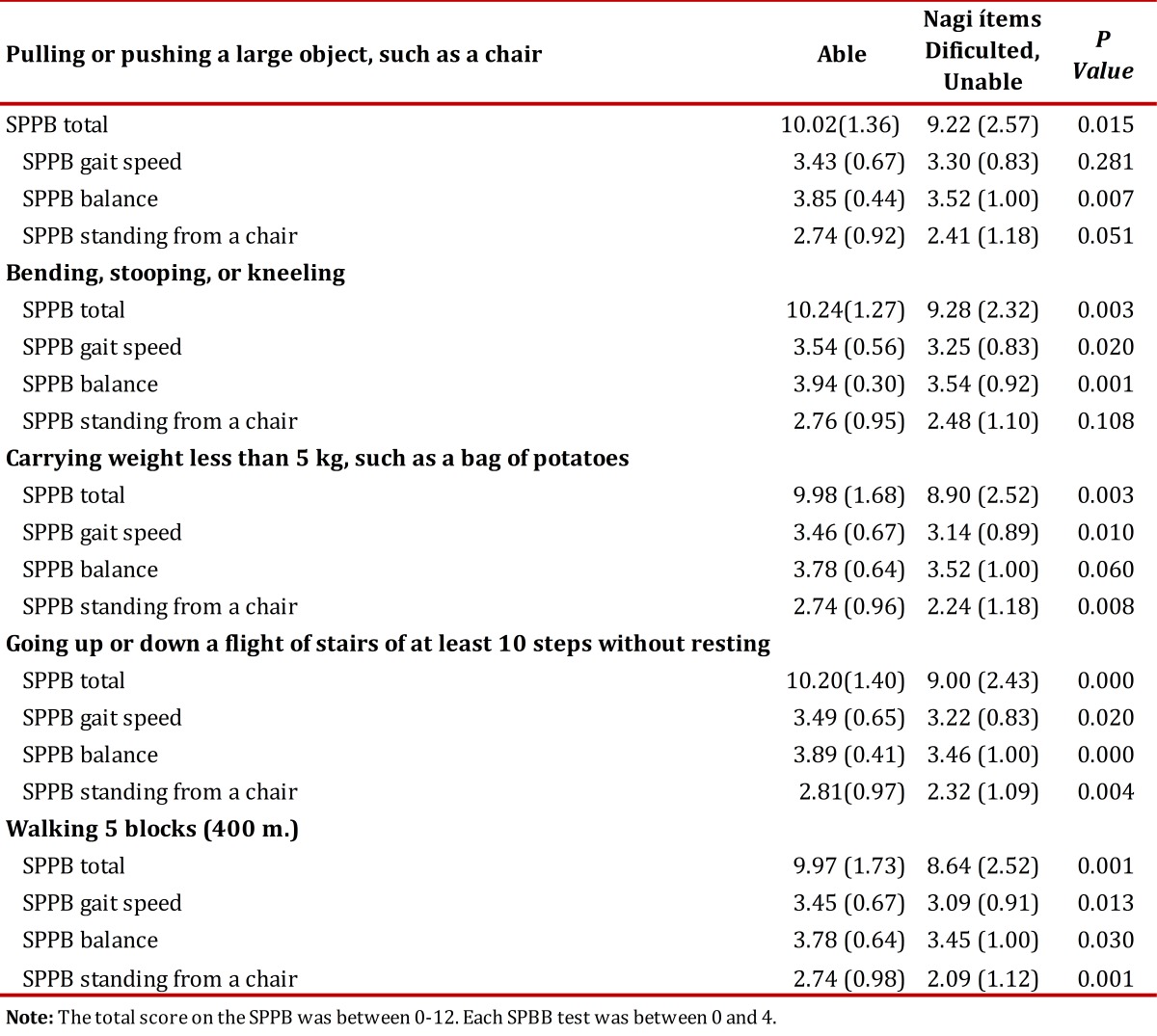
Average scores (SD) of the total SPPB battery & for each test according to difficulties on the Nagi Scale

**Table 5:**
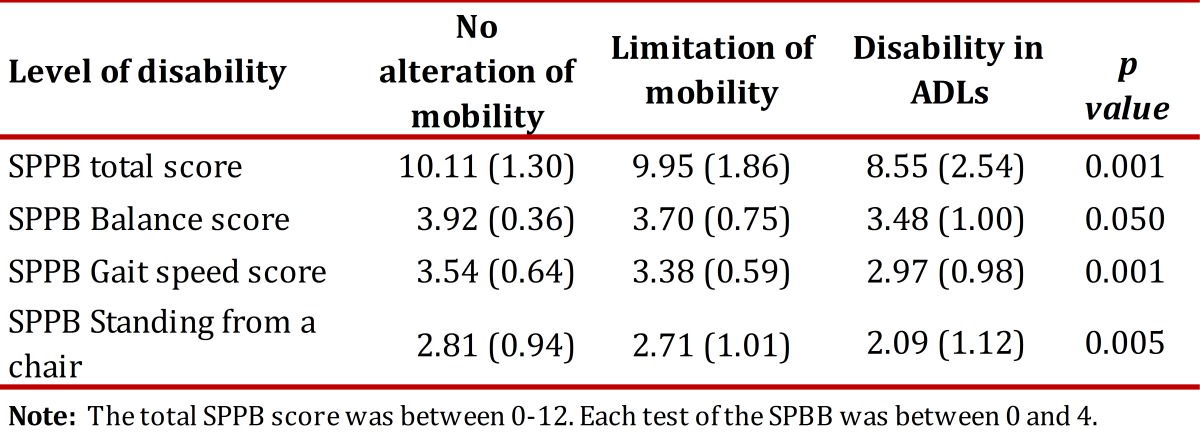
Average (SD) of the total score and of each test of the short physical performance battery (SPPB) according to level of disability.

## Discussion

In this study we examined the ability of the SPPB to assess physical performance in elderly Colombians from the Andean region. The main findings can be summarized as follows: the SPPB is a valid and reliable instrument to evaluate the mobility of the elderly Colombians. The validity of the SPPB was demonstrated by the relationship found with measures of health status and variables of functional capacity.

The test-retest reliability was high, 0.87, a result that is consistent with other studies. In U.S. populations, a within group correlation coefficient was found between 0.88 and 0.92^11^ ^,^ ^14^. In the study comparing Brazilian and Canadian populations, the intra-observer reliability in Brazil was 0.83 (95%CI, 0.73-0.89) and in Canada it was 0.89 (95% CI, 0.83-0.93)^16^. Besides the reported test-retest reliability in a sample of 30 elderly institutionalized Brazilians was 0.88^21^. In elderly Spanish from five primary care centers, the test-retest reliability correlation ranged from 0.6 (95%CI: 0.35-0.70) for the balance test and 0.8 (95%CI: 0.67 to 0.86) for test of gait speed^19^. Our results are consistent with these findings.

Regarding convergent validity, our results are related to those reported for similar populations. For example, the validation study conducted with elderly Canadians and Brazilians and the results of this study confirm the finding that the SPPB battery is an objective measure of physical performance applicable to different cultures, different social groups and to different languages^16^. As in previous studies, we found that the SPPB battery is related to functional and mobility factors^2^ ^,^ ^10^ ^,^ ^22^. In this regard, other studies have shown that physical performance measures predict difficulties with ADLs^2^ ^,^ ^13^, and loss of the ability to walk 400 m in the next 3 years^23^. The SPPB battery is an excellent predictor of the beginning of difficulties in physical ADLs at 12 months^14^. On the other hand, a recent article by several authors from this study showed that the SPPB battery is useful also for identifying frail elderly people in different socioeconomic contexts^24^. Thus, the findings regarding the references make the SPPB bacteria an important tool in the identification of the loss of mobility in the elderly.

In our study, the total score of the SPPB and the walking speed were more consistently related to the disability level, a finding similar to that reported in the literature^2^ ^,^ ^25^. A speed <0.6 m/s in the 4 m test is considered a cutoff point for identifying persons at high risk of being hospitalized with deteriorating health and physical function^4^. In this study, a gait speed of 0.6 m/s corresponded to 6 points on the SPPB, results similar to those reported by Cabrero-Garcia *et al* ^19^. With the gait speed found in our study, 8.1% of the population would be at risk of an adverse effect, unlike other studies that reported 19.6% of the population at risk^19^.

Regarding construct validity, the SPPB battery was related to variables of self perceived health, cognitive function and depression in a manner consistent with that observed in the scientific literature^9^ ^,^ ^16^ ^,^ ^17^ ^,^ ^21^. In a recent study conducted in Brazil, a close relationship was shown between the total score for the SPPB and self perceptions of health, as was the case in this study^22^. Also, as with our own results, in the Canadian-Brazilian validation study, a gradual decrease was observed in the average total scores for the SPPB battery in the measure of increasing limitations with the lower limbs and with disability^16^. In another validation study of the SPPB in primary care of Spanish elderly over 70 years of age, it was found that the SPPB battery was associated with statistically significant differences in dependency with physical and instrumental ADLs, depression and self-perceptions of health^19^, as was similarly found in this study.

Our study has several limitations. First, due to its cross-sectional design, it was not possible to evaluate the predictive validity of the battery. Second, given the convenience sampling type, it was not possible to generalize our results to the entire population. Despite these limitations, this study contributes to establishing the validity and reliability of this instrument for physical performance measurement.

Although the use of SPPB battery is still limited in clinical settings by the perception that it requires a large space, sophisticated equipment and training, and that it takes extended periods of time to implement^4^, the SPPB battery took no more than 5 minutes to carry out and could be applied in any clinical setting^19^, even by non-specialized personnel, as it only requires appropriate training in its application. In addition, several studies have shown that its use is feasible in primary care units^13^. Another aspect to consider for increasing its use is the clinical significance of the results: non-disabled elderly who score below 10 on the battery will have a high risk of developing disability in the future^23^.

In conclusion, our study shows that the SPPB battery is a valid and reliable instrument to assess physical performance in the elderly, as well as being safe and easy to administer. Its potential use to measure mobility limitation and disability in a broad spectrum of physical functionality makes it a battery that should be considered both in clinical practice and in longitudinal research.
